# Optical biosensor differentiates signaling of endogenous PAR_1 _and PAR_2 _in A431 cells

**DOI:** 10.1186/1471-2121-8-24

**Published:** 2007-06-22

**Authors:** Ye Fang, Ann M Ferrie

**Affiliations:** 1 Biochemical Technologies, Science and Technology Division, Corning Incorporated, Sullivan Park, Corning, NY 14831, USA

## Abstract

**Background:**

Protease activated receptors (PARs) consist of a family of four G protein-coupled receptors. Many types of cells express several PARs, whose physiological significance is mostly unknown.

**Results:**

Here, we show that non-invasive resonant waveguide grating (RWG) biosensor differentiates signaling of endogenous protease activated receptor subtype 1 (PAR_1_) and 2 (PAR_2_) in human epidermoid carcinoma A431 cells. The biosensor directly measures dynamic mass redistribution (DMR) resulted from ligand-induced receptor activation in adherent cells. In A431, both PAR_1 _and PAR_2 _agonists, but neither PAR_3 _nor PAR_4 _agonists, trigger dose-dependent Ca^2+ ^mobilization as well as G_q_-type DMR signals. Both Ca^2+ ^flux and DMR signals display comparable desensitization patterns upon repeated stimulation with different combinations of agonists. However, PAR_1 _and PAR_2 _exhibit distinct kinetics of receptor re-sensitization. Furthermore, both trypsin- and thrombin-induced Ca^2+ ^flux signals show almost identical dependence on cell surface cholesterol level, but their corresponding DMR signals present different sensitivities.

**Conclusion:**

Optical biosensor provides an alternative readout for examining receptor activation under physiologically relevant conditions, and differentiates the signaling of endogenous PAR_1 _and PAR_2 _in A431.

## Background

Protease activated receptors (PARs) comprise a family of G protein-coupled receptors (GPCRs) which to date include PAR_1_, PAR_2_, PAR_3 _and PAR_4 _[1-5]. Instead of being activated through reversible ligand binding, PARs utilize a unique proteolytic mechanism for activation [6-8]. Serine proteases such as thrombin and trypsin site-specifically cleave the receptor within the extracellular N-terminal exodomain. The activating cleavage site is the residue 41–42 (R↓SFLLRN), 36–37(R↓SLIGKV), 38–39 (K↓TFRGAP) and 47–48 (R↓GYPGQV) for human PAR_1_, PAR_2_, PAR_3 _and PAR_4_, respectively [9]. The cleavage unmasks a new N-terminus, which, in turn, acts as a tethered ligand sequence. The tethered ligand domain binds intramolecularly to and activates the receptor, thus initiating signaling. The proteases that activate PARs include coagulation factors (e.g. thrombin, coagulation factors VIIa and Xa), proteases from inflammatory cells (e.g., mast cell tryptase, neutrophil cathepsin G) and enzymes from epithelial tissues (e.g., trypsins). PAR_1_, PAR_3 _and PAR_4 _are activated principally by thrombin, while PAR_2 _is activated by trypsin-like proteases such as mast cell tryptase and coagulation Factor Xa. Synthetic PAR-activating peptides (PAR-APs), corresponding to the first five or six amino acids of the tethered ligand sequences, can directly activate PARs, except for PAR_3 _[3,9-13]. Since these synthetic peptides function as receptor agonists independent of proteolysis, PAR-APs are useful for studying the physiological and pathophysiological functions of PARs.

PARs are found in a large variety of normal and malignant tissues and cells including skin, platelets, endothelial cells, gastrointestinal tract, brain and lungs. Most cell types express several PARs; for example, A431 cells endogenously express PAR_1 _and PAR_2 _[14,15]. The presence of several PARs in a cell type makes it unclear how the cell differentiates among these signaling. Recently, we had developed a non-invasive and manipulation-free cell assay technology, termed MRCAT (Mass Redistribution Cell Assay Technology), which is centred on resonant waveguide grating (RWG) biosensor [16]. The RWG biosensor directly measures ligand-induced dynamic mass redistribution (DMR) within the bottom portion of adherent cells. Theoretical and numerical analysis suggests that the resultant DMR signal such as the G_q_-type DMR [16] represents a novel physiological readout for cell signaling, which consists of contributions of many cellular events downstream the ligand-induced receptor activation. The MRCAT enables the study of systems cell biology of receptors including epidermal growth factor receptor (EGFR) [17] and bradykinin B_2 _receptor [18]. Here we applied the MRCAT to investigate the actions of several PAR agonists, with a special emphasis on the roles of cholesterol in regulating the signaling of endogenous PARs in A431 cells.

## Results

### PAR_1 _and PAR_2 _transduce G_q/11 _signaling in A431

**Figure 1:**
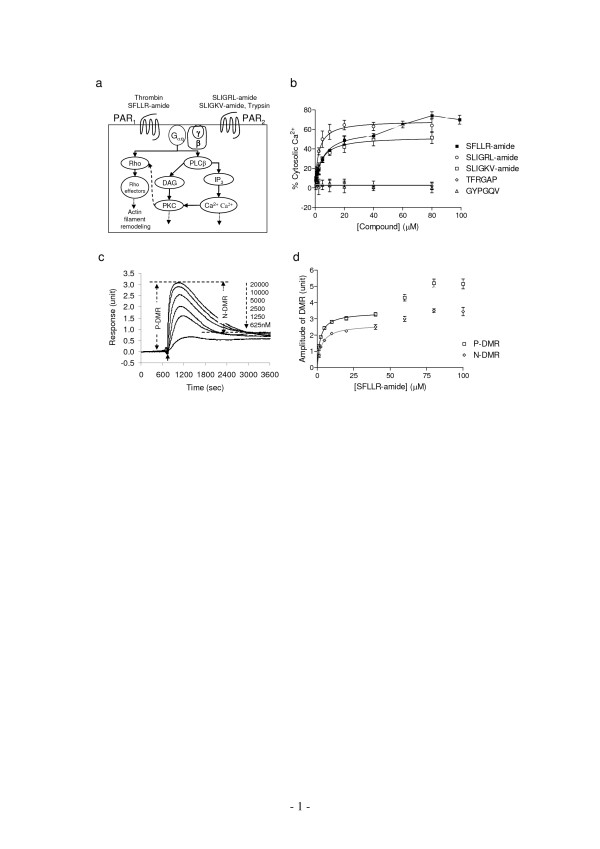
**The Ca^2+ ^mobilization and DMR signals mediated through endogenous PARs in A431**. (a) The endogenous PARs and their corresponding agonists. Both receptors mediate G_q _signaling, which proceeds through activation of the receptor, its coupled G protein and downstream target phospholipase C (PLC). The PLC hydrolyzes the membrane lipid phosphatidylinositol bisphosphate (PIP_2_), producing inositol triphosphate (IP_3_) and diacylglycerol (DAG). IP_3 _binds to and opens a calcium channel in the endoplasmic reticulum, leading to calcium mobilization. Calcium alters many cellular processes. The interaction of both DAG and calcium with protein kinase C (PKC) activates PKC kinase activity, which, in turn, phosphorylates many different protein targets including small GTPase Rho, leading to the remodeling of cytoskeletal structure. (b) The increase in intracellular Ca^2+ ^level as a function of the concentration of different soluble PAR agonists. (c) The real-time dynamic mass redistribution signals induced by SFLLR-amide at different doses. The solid arrow indicates the time when SFLLR-amide is introduced. The DMR consists of two phases: an increase signal (termed Positive-DMR, P-DMR) and a sequential decay signal (termed Negative-DMR, N-DMR). (d) The amplitudes of both P-DMR and N-DMR events, calculated as indicated in (c), as a function of SFLLR-amide concentration.

**Figure 2:**
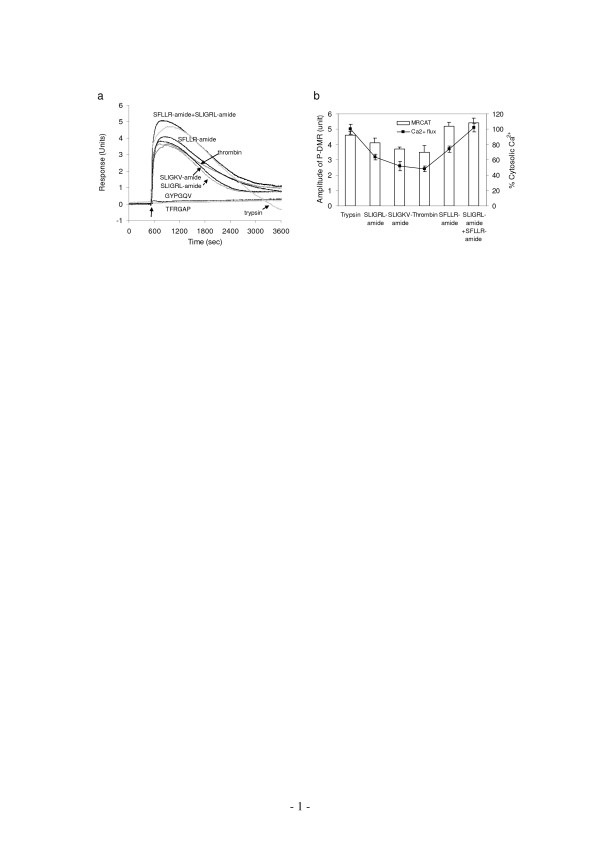
**Correlation between the maximal Ca^2+ ^mobilization and DMR responses induced by PAR agonists**. (a) The DMR signals induced by PAR agonists: TFRGAP (20 μM), GYPGQV (20 μM), SLIGRL-amide (20 μM), SLIGKV-amide (20 μM), thrombin (40 unit/ml), SFLLR-amide (20 μM), trypsin (1024 nM), SFLLR-amide+SLIGRL-amide (each at 20 μM). (b) Comparison of the maximal DMR and Ca^2+ ^mobilization responses induced by different PAR agonists. The DMR response was calculated using the amplitude of the P-DMR event. Since trypsin at doses greater than ~1000 nM led to significant cell detachment (ref. 16), the DMR signal induced by trypsin at 1024 nM was used as its maximal response.

To probe the signaling of endogenous PARs in A431, both conventional Ca^2+ ^flux assay and the MRCAT were used to examine the cellular responses induced by several PAR agonists. The agonists were thrombin and SFLLR-amide (PAR_1_), trypsin, SLIGKV-amide and SLIGRL-amide (PAR_2_), TFRGAP (PAR_3_), and GYPGQV (PAR_4_). Among them, only PAR_1 _and PAR_2 _agonists resulted in rapid and transient increase in intracellular Ca^2+ ^([Ca^2+^]_i_) as well as G_q_-type DMR signals in quiescent A431 cells (Fig. 1 and Fig. 2a). These results were consistent with the expression pattern and signaling of endogenous PARs in A431. A431 is known to express PAR_1 _and PAR_2_, which both mediate classical G_q _signaling [14,15].

Both Ca^2+ ^mobilization and DMR signals were dose-dependent and saturable to each agonist, including trypsin or SFLLR-amide at low doses. The saturation curves obtained seem fit well with one-site binding, based on non-linear regression and Scatchard analysis. The EC_50 _values of each agonist obtained using both methods were comparable (Table 1), suggesting that similar to conventional Ca^2+ ^signals, the ligand-induced DMR signals could also be used as alternative readouts for examining receptor activation. We previously had shown that trypsin of high doses (>~1000 nM) led to complicated DMR signals, due to the combination of trypsin-induced cell signaling and cell detachment from the sensor surface [16]. Thus, the DMR signals induced by trypsin only at low doses were analyzed. Interestingly, SFLLR-amide at high doses (>40 μM) led to intracellular Ca^2+ ^level as well as DMR signal to another elevated level (Fig. 1b and 1d). A possible explanation is that SFLLR-amide at high doses activates both PAR_1 _and PAR_2_, consistent with the previous findings done by others that SFLLR-amide has higher efficacy to activate PAR_1 _than PAR_2 _[9]. Alternatively, beside the G_q _pathway, SFLLR-amide at high doses may also lead to the activation of a second signaling pathway, which could further increase both the Ca^2+ ^mobilization and DMR signals. It has been reported that the activation of several G_i_-coupled receptors results in Ca^2+ ^mobilization through G_βγ _subunits of G_i/o _proteins [19], and a dose-dependent switching of receptor signaling could occur for some GPCR-ligand systems [20].

**Table 1:** EC_50 _values of PAR agonists in A431. EC_50 _values were obtained using conventional Ca^2+ ^flux assay, in comparison with those obtained using the MRCAT. In the case of MRCAT data, the amplitudes of both P-DMR and N-DMR events as a function of agonist concentration (as indicated in Fig. 1c) were used to calculate EC_50_.

**Ligand**	**EC_**50 **_(n = 3)**		
	
	**Ca^**2+ **^flux assay**	**P-DMR**	**N-DMR**
Trypsin	45.7 ± 5.8 nM	98.0 ± 27.8 nM	102.1 ± 21.9 nM
SLIGRL-amide	2.5 ± 0.3 μM	2.3 ± 0.6 μM	3.2 ± 0.8 μM
SLIGKV-amide	3.8 ± 0.4 μM	6.1 ± 1.0 μM	9.1 ± 2.2 μM
Thrombin	6.0 ± 1.0 unit/ml	9.6 ± 2.0 unit/ml	11.0 ± 1.9 unit/ml
SFLLR-amide	5.0 ± 0.4 μM	1.9 ± 0.1 μM	3.1 ± 0.2 μM

### Functional interactions between PAR_1 _and PAR_2_

Since A431 expresses both PAR_1 _and PAR_2_, we were interested in the possibility of functional interactions between the two receptors. First, we examined the maximal responses induced by PAR agonists (Fig. 2). The maximal [Ca^2+^]_i _elevation induced by trypsin, SLIGRL-amide, SLIGKV-amide, thrombin, and SFLLR-amide was found to be 100 ± 6%, 64 ± 4%, 52 ± 6%, 48 ± 4%, and 74 ± 4%, respectively. The maximal [Ca^2+^]_i _elevation induced by trypsin was approximately 2 fold as high as those induced by thrombin, SLIGKV-amide or SLIGRL-amide, whereas SFLLR-amide at 80 μM led to an intermediate maximal [Ca^2+^]_i _elevation. Furthermore, a mixture of SFLLR-amide and SLIGKV-amide (both at 20 μM) resulted in an [Ca^2+^]_i _elevation of 102 ± 5.4%. At saturating concentrations, these agonists also led to similar DMR signals but with different maximal amplitudes (Fig. 2a). The maximal amplitudes of these DMR signals had an order that is almost identical, but less pronounced, to those obtained using Ca^2+ ^flux measurements (Fig. 2b). It is worthy noting that the PAR_2_-specific agonist SLIGKV-amide led to relatively lower maximal responses, in terms of both Ca^2+ ^mobilization and DMR signal, than another PAR_2_-specific agonist SLIGRL-amide did.

**Figure 3:**
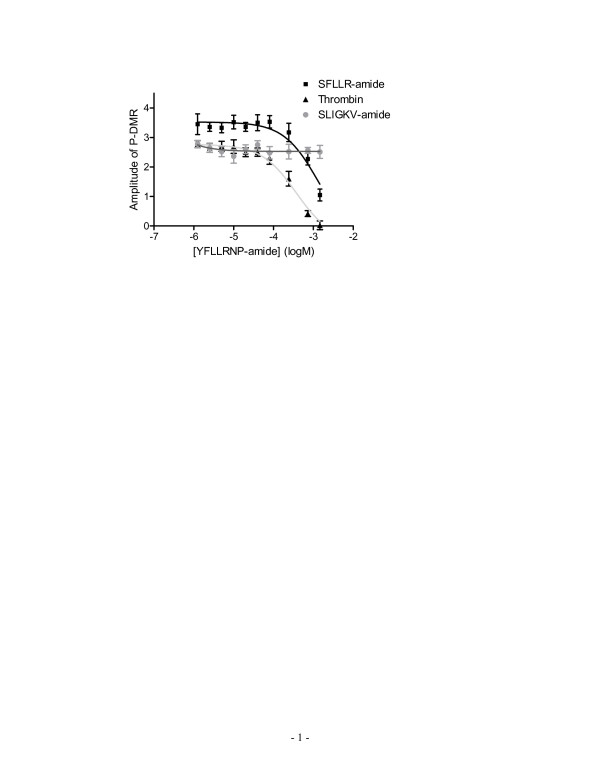
**The effect of YFLLRNP-amide on the DMR signals induced by PAR agonists**. The cells were pre-treated with YFLLRNP-amide at different doses. The amplitudes of the P-DMR events induced by each agonist (40 unit/ml thrombin, 20 μM SFLLR-amide, or 20 μM SLIGKV-amide) were plotted as a function of YFLLRNP-amide concentration.

Second, we examined the effect of PAR_1 _specific partial agonist YFLLRNP-amide on ligand-induced cellular responses. Results showed that YFLLRNP-amide at doses up to 729 μM did not lead to any significant elevation of [Ca^2+^]_i_; however, at high doses (>80 μM) it led to a small DMR signal (data not shown). This is consistent with the previous observations done by others that YFLLRNP-amide can activate G_12/13_resulting in the cell shape change, but not G_q_-mediated Ca^2+ ^signaling in human platelets [21,22]. Furthermore, YFLLRNP-amide dose-dependently attenuated the DMR signals induced by thrombin or SFLLR-amide, but not SLIGKV-amide (Fig. 3). However, at the highest dose examined YFLLRNP-amide almost completely blocked the thrombin-induced DMR, but only partially attenuated the SFLLR-amide-induced DMR.

**Figure 4:**
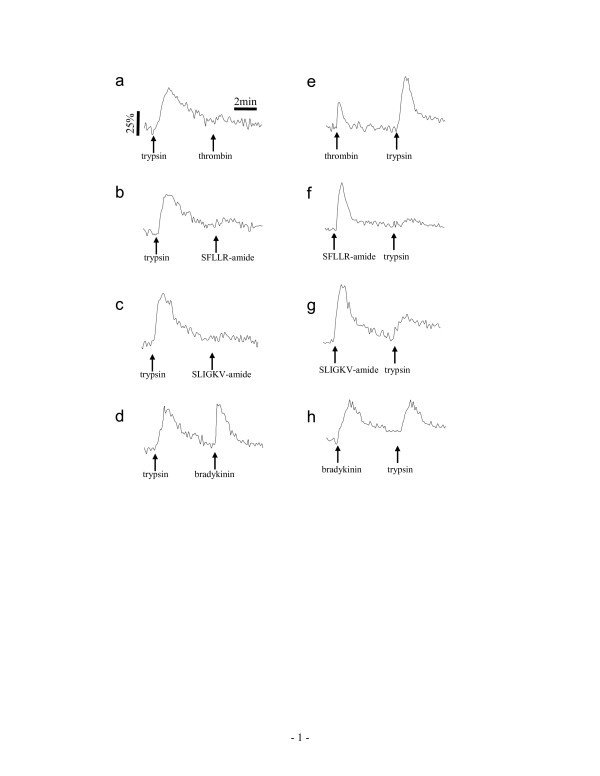
**Desensitization of A431 cells to repeated agonist stimulation – Ca^2+ ^mobilization**. The cells were subject to repeated stimulation, separated by 6 min, with various combinations of agonists. The agonist concentration was 40 unit/ml, 200 nM, 20 μM, 20 μM, and 100 nM for thrombin, trypsin, SFLLR-amide, SLIGKV-amide, and bradykinin, respectively.

Third, we examined the desensitization patterns of receptor signaling, since either thrombin or trypsin potentially activates several PARs. Figure 4 summarized Ca^2+ ^mobilization patterns in A431 in response to repeated stimulation, separated by 6 min, with various combinations of agonists. Pre-stimulation of A431 with trypsin completely blocked the ability of PAR agonists (thrombin, SFLLR-amide, SLIGKV-amide, SLIGRL-amide, or trypsin) to provoke Ca^2+ ^moblization (Fig. 4a–c, data not shown). Conversely, neither of PAR agonists examined had any effect on the Ca^2+ ^mobilization induced by bradykinin, an agonist for bradykinin B_2 _receptor which is also expressed in A431 [18,23] (exampled in Fig. 4d), suggesting that the inhibition of the response to PAR agonists by the preceding stimulation with trypsin is not due to any non-specific digestion of the membrane proteins. On the other hand, the pre-stimulation with thrombin completely inhibited the responsiveness of cells to thrombin, only partially to trypsin, but not to SLIGKV-amide or SLIGRL-amide. In addition, the preceding stimulation with SFLLR-amide, SLIGKV-amide or SLIGRL-amide all partially attenuated the trypsin-induced Ca^2+ ^mobilization (Fig. 4e–g; data not shown). Conversely, SFLLR-amide still led to a significant intracellular Ca^2+ ^elevation (32 ± 4.3%) in the thrombin-stimulated cells, consistent with the previous observations done by others that SFLLR-amide activates both PAR_1 _and PAR_2 _[24,25].

**Figure 5:**
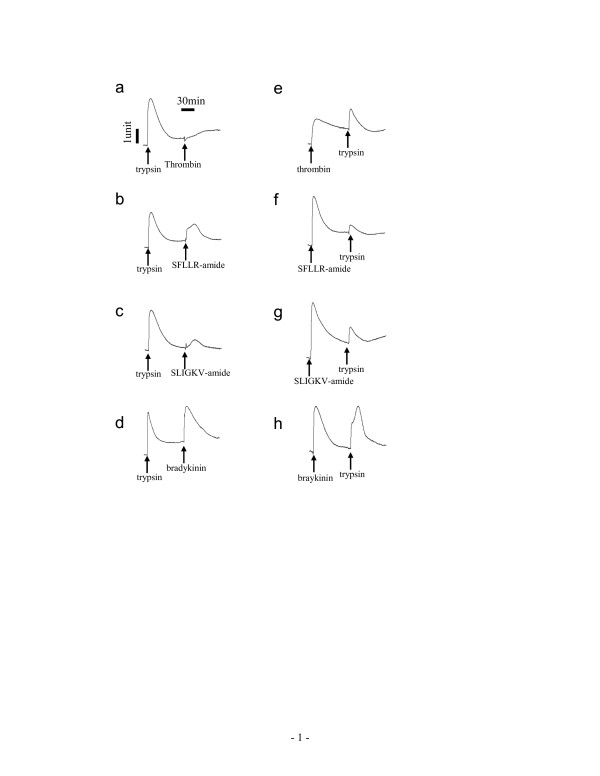
**Desensitization of A431 cells to repeated agonist stimulation – DMR signal**. The cells were subject to repeated stimulation, separated by ~1 hr, with various combinations of agonists. The agonist concentration was 40 unit/ml, 200 nM, 20 μM, 20 μM, and 100 nM for thrombin, trypsin, SFLLR-amide, SLIGKV-amide, and bradykinin, respectively.

Since the ligand-induced G_q_-type DMR signals typically proceed about 30 min to 1 hour, we were interested in the desensitization and resensitization patterns of cells in response to repeated stimulation, separated by ~1 hr, with various combinations of GPCR agonists. Results showed that the trypsin-treated cells became completely desensitized to trypsin, or thrombin (Fig. 5a), only partially to SFLLR-amide, SLIGKV-amide, or SLIGRL-amide (Fig. 5b–c, data not shown), but not to bradykinin (Fig. 5d). On the other hand, the preceding stimulation with thrombin, SFLLR-amide, SLIGKV-amide or SLIGRL-amide only partially attenuated the DMR signal induced by trypsin (Fig. 5e–g; data not shown), while bradykinin had no obvious effect on the DMR signal induced by any PAR agonist (Fig. 5h, data not shown). Furthermore, SLIGKV-amide and SLIGRL-amide led to almost identical DMR signals in cells with or without pre-stimulation with thrombin (data not shown). Together, these results suggest that both SFLLR-amide and trypsin may activate both PAR_1 _and PAR_2_.

### Differentiated regulation of PAR_1 _and PAR_2 _signaling

**Figure 6:**
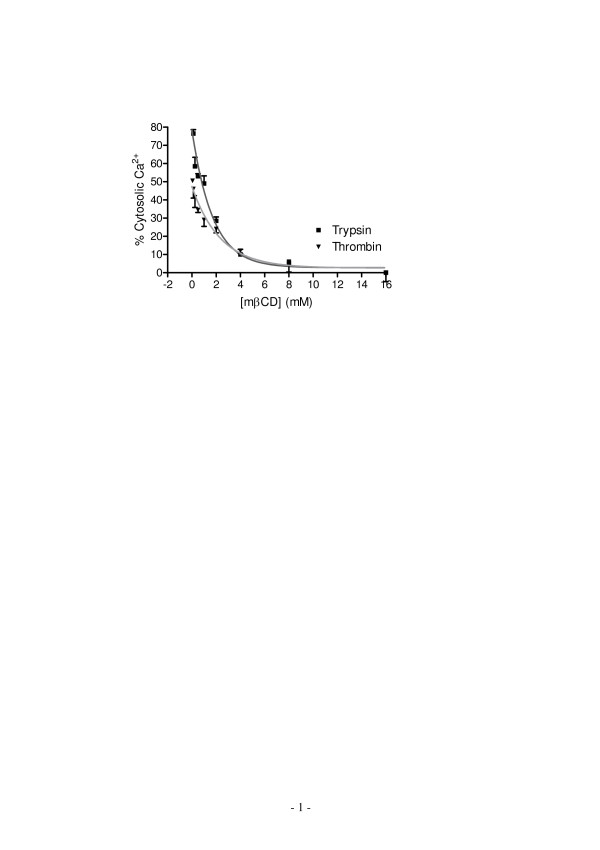
**Cholesterol removal impairs the agonist-induced Ca^2+ ^mobilization**. mβCD was used to deplete cell surface cholesterol. Its effect on agonist-induced Ca^2+ ^mobilization was examined. The agonists were trypsin (200 nM) and thrombin (40 unit/ml).

Since the physiological significance of co-expression of two receptors in A431 is unclear, we were interested in the possibility of differentiated regulation of PAR_1 _and PAR_2 _signaling. A common mechanism for the cell to regulate and differentiate receptor signaling is compartmentalization of receptors and/or their signaling components. Amassing evidences support the hypothesis that there are many cholesterol-rich microdomains at the cell surface membrane, although their characteristics are still under extensive investigations. Such microdomain is believed to enable the sorting and segregation of receptors and their signaling cascades, thus regulating cell signaling [26]. Methyl-β-cyclodextrin (mβCD) has been widely used to extract cell surface choleterol primarily through effluxing [27], thus disrupting these cholesterol-rich microdomains. Thus, we examined the effect of cholesterol depletion by mβCD on both Ca^2+ ^mobilization and DMR responses induced by thrombin and trypsin. The pre-treatment of quiescent A431 cells with mβCD up to 16 mM led to a dose-dependent suppression of Ca^2+ ^mobilization induced by either trypsin or thrombin (Fig. 6). Both dose-dependent inhibition curves appeared fit well with a one-phase decay non-linear regression, leading to an almost identical half-concentration (C_1/2 _of ~1.5 mM) of mβCD. This is consistent with the fact that mβCD results in rapid effluxing of cell membrane cholesterol molecules; and the amount of remain cell surface cholesterol depends on the concentration of mβCD in solution [27]. The higher concentration mβCD is, the less cholesterol is retained at the cell surface membrane. Since α-cyclodextrin (αCD) is an inactive cyclodextrin analog and has been found to be incapable of extracting cholesterol from cultured cells [28], αCD was used as a negative control to study the regulation of PAR signaling by cholesterol. Unlike mβCD up to 16 mM, αCD at high doses (>8 mM) resulted in detectable cell toxicity as well as significant amounts of cells detached from the biosensor surface (data not shown). Thus, only low doses of αCD were used. Results showed that αCD up to 8 mM had little effect on both trypsin and thrombin-induced Ca^2+ ^mobilization responses (data not shown).

**Figure 7:**
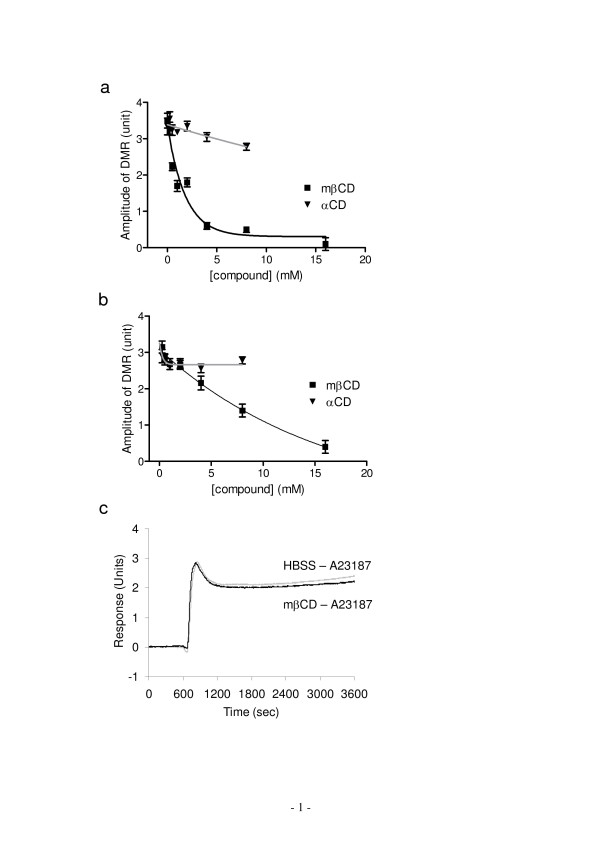
**Cholesterol removal attenuates the agonist-induced DMR signals**. mβCD was used to deplete cell surface cholesterol. Its effect on the amplitudes of the P-DMR events induced by (a) thrombin (40 unit/ml) or (b) trypsin (200 nM) was analyzed. In comparison, the effect of αCD was also included. (c) The DMR signals of A431 cells induced by A23187 without or with the pre-treatment with 5 mM mβCD.

Similarly, the mβCD pre-treatment also led to a dose-dependent attenuation of the DMR signals induced by thrombin or trypsin (Fig. 7). However, there is a clear distinction between the inhibition curves of trypsin- and thrombin-induced DMR signals by mβCD. The suppression of the thrombin-induced DMR by mβCD exhibited a dose-dependency (C_1/2 _of 1.2 ± 0.3 mM) similar to that measured with Ca^2+ ^mobilization (Fig. 7a). On the other hand, the inhibition of the trypsin-mediated DMR by mβCD displayed a much slower decay to increased concentrations of mβCD, which apparently fits well with a 2-phase decay, leading to C_1/2 _of 5 ± 1 mM and 11 ± 2 mM, respectively (Fig. 7b). This difference suggests that the trypsin-mediated DMR signal involves more complicated cellular mechanisms than that induced by thrombin. As expected, αCD up to 8 mM had little effect on both agonist-induced DMR responses. On the other hand, the pre-treatment of A431 cells with mβCD at 5 mM had little effect on the DMR signal induced by 5 μM A23187 (Fig. 7c), suggesting that the cholesterol-depleted cells are still responsive to non-membrane elicited events. A23187 is a Ca^2+ ^ionophore, and is able to release stored Ca^2+ ^from the endoplasmic reticulum in cells. Interestingly, the A23187-induced DMR signal somewhat mimics the PAR agonist-induced DMR signals (Fig. 2a), suggesting that Ca^2+ ^pathway is part of the response pathway(s), and the PAR agonist-induced DMR is largely downstream cellular events of Ca^2+ ^mobilization mediated through the receptor activation.

**Figure 8:**
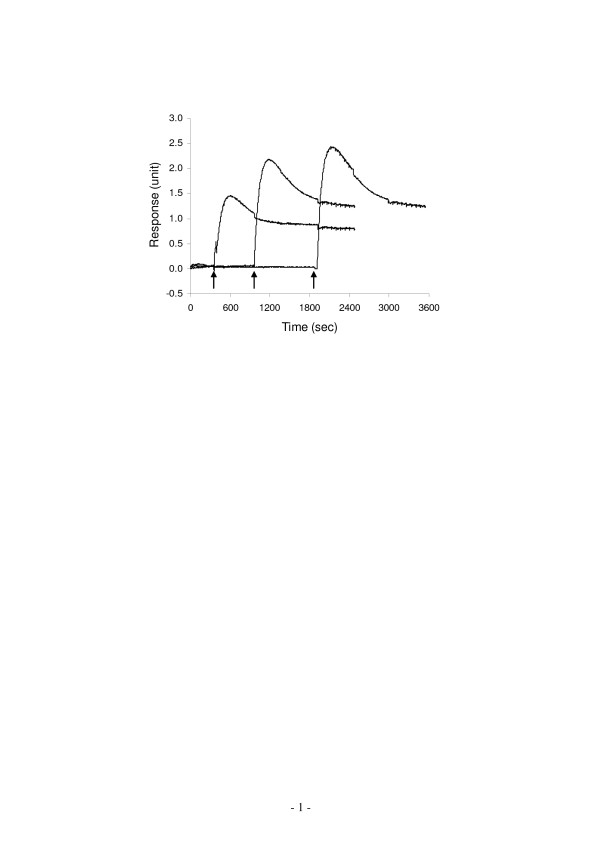
**The functional recovery of PAR signaling after cholesterol removal**. mβCD was used to extract cell surface cholesterol. After the mβCD-containing medium was replaced with the DMEM, thrombin was introduced to stimulate cells at different time. Each graph is an average of 7 independent responses.

We further studied the functional recovery of PAR signaling after cholesterol depletion with mβCD. This was based on the timely recovery of cell surface cholesterol in the mβCD-treated cells after replacing the medium containing mβCD with the medium only. Results showed that the thrombin-induced DMR signal progressively recovered (Fig. 8), indicating that the formation of cholesterol-assisted microdomains is dynamic and reversible, and cholesterol concentration at the cell membranes is important in regulating the PAR signaling.

**Figure 9:**
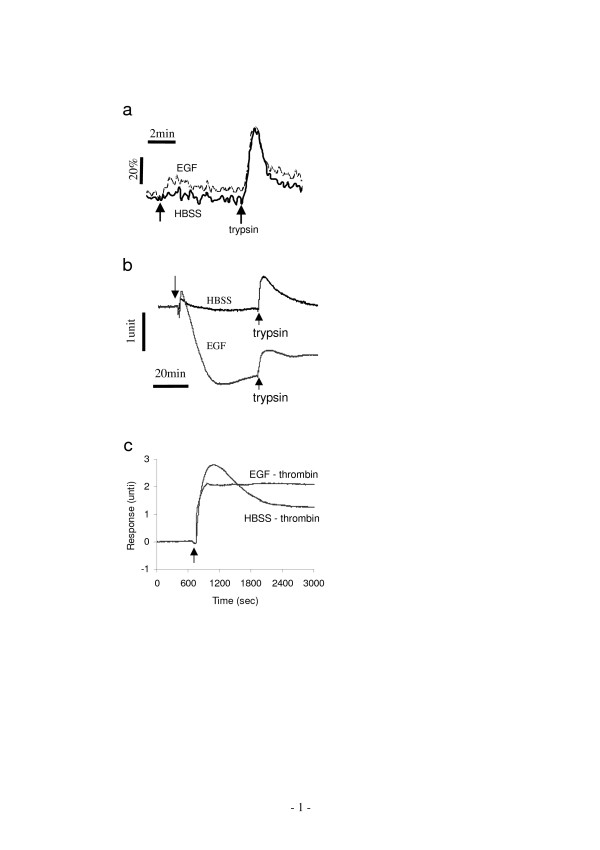
**The effect of preceding EGF stimulation on PAR signaling**. (a) The Ca^2+ ^mobilization. (b, c) The DMR signals. The ligands were EGF (100 nM), trypsin (100 nM), and thrombin (40 unit/ml). The cell responses with the pre-treatment with the HBSS only were also included as control.

Previously we had shown that blockage of EGFR tyrosine kinase activity by AG1478 partially attenuated the trypsin-induced DMR signal in A431 [16], suggesting that EGFR transactivation may be a downstream event of trypsin-induced response. A431 cells express large numbers of EGF receptors [29]. AG1478 is a potent and selective EGFR tyrosine kinase inhibitor. It is also known that cholesterol removal by mβCD triggers a ligand-independent transactivation of EGFR in A431 cells [28,30]. Thus, we were interested the interference of EGFR activation with PAR signaling. The preceding stimulation with EGF had little effect on the Ca^2+ ^mobilization induced by trypsin (Fig. 9a) or thrombin (data not shown). However, at 100 nM EGF almost completely inhibited the N-DMR event, but only slightly attenuated the P-DMR event in the DMR signal induced by either trypsin (Fig. 9b) or thrombin (Fig. 9c). However, the pre-treatment of A431 cells with AG1478 did not counter the inhibitory effect of cholesterol depletion on the trypsin-induced DMR signals (data not shown). Together, these results suggest that both trypsin and thrombin-induced signaling are sensitive to EGFR activation.

## Discussion

There is growing evidence that GPCR signaling is complicated – many GPCRs including PAR_2 _elicit both G protein-dependent and independent signaling events [20,31]. To complicate this, a GPCR may exist in a collection of microstates (i.e., conformations), and different agonists may result in distinct active-state conformations, thus directing the receptor-induced signals to various cellular pathways [32,33]. Conventional cell-based assays typically measure a singular cellular response (e.g., second messenger generation, protein interactions or trafficking). Because of that, these assays may lead to false negatives, due to alternative pathway a ligand may selectively activate. On the other hand, the non-invasive optical biosensor used here utilizes an evanescent wave with a short penetration depth (~200 nm) to probe ligand-induced dynamic redistribution of cellular matter; the resultant DMR signal is an integrated cellular response [16]. Thus, the use of the DMR signal as an alternative readout for receptor activation is advantageous. Since many cell signaling events involve significant redistribution of cellular matters, the biosensor-based assays may find broad applicability in many different types of targets including GPCRs [16,18,34], EGFR [17] and ion channels (unpublished data). In A431 cells, we recently have identified three classes of DMR signals induced by panels of GPCR agonists targeting several endogenous receptors, each of which was correlated well with the activation of a class of GPCRs, depending on the G protein with which the receptor is coupled (i.e., G_q_, G_s _and G_i_).

Because of the short penetration depth of the evanescent wave of the present biosensor, only cellular events occurring within the detection zone of the cell layer contribute to ligand-induced DMR signals. Although ligand-induced receptor activation may lead to an array of signaling pathways or events [16-18], only signaling events having significant redistribution of cellular matters could be detected. Recently we have developed a mathematical model for the DMR signals mediated by G_q_-coupled receptors [16]. We also have shown that a GPCR ligand-induced DMR signal primarily consists of three components: trafficking of intracellular targets to the activated receptors and subsequently receptor internalization [16], changes in cell adhesion degree [35], and cytoskeletal remodelling which is at the crossroad of the receptor activation to downstream signaling events [[18], unpublished data].

Here we examined the signaling of endogenous PARs in A431. Among PAR agonists examined, only PAR_1 _and PAR_2 _agonists resulted in significant Ca^2+ ^mobilization and G_q_-type DMR signals. This result is consistent with the fact that only PAR_1 _and PAR_2 _are endogenously expressed in A431, and both receptors elicit G_q _signaling. The DMR signals induced by all PAR_1 _and PAR_2 _agonists share almost identical dynamics, except of the signaling amplitudes (Fig. 2a). The overall dynamics – an initial rapid P-DMR event followed by a relatively slow N-DMR event – is also similar to those mediated through the activation of other endogenous G_q_-coupled receptors in A431 cells [[17,34]; unpublished data]. Our recent theoretical analysis suggests that for G_q_-coupled receptors, the P-DMR is primarily resulted from the recruitment of intracellular targets to the activated receptors at the cell membrane, while the receptor internalization is a major contributor to the N-DMR event [16]. Furthermore, the PAR agonist-induced DMR signals also share similarity with the Ca^2+ ^ionophore A23187-induced DMR signal (Fig. 7c), suggesting that the DMR signals of PAR agonists obtained are mainly downstream of Ca^2+ ^pathway. In addition, compared to Ca^2+ ^mobilization signals, the less pronounced difference in the maximal DMR responses of different PAR agonists also indicates that the DMR signals are downstream of Ca^2+ ^mobilization. It is a recent finding that in many cases an agonist-induced maximal response, a measure of agonist efficacy, is dependent on the cellular events measured. This is because GPCR signaling typically proceeds through a series of amplification steps. As a result, the closer to the receptor activation step the cellular event measured is, the bigger difference the agonist efficacy might be [32]. Together, these results suggest that the DMR signals of PAR agonists are primarily resulted from G_q _signaling, although one cannot rule out the contributions of other signaling pathways to the overall DMR signal.

Nonetheless, similar to Ca^2+ ^mobilization, the ligand-induced DMR signals are not only dependent on and saturable to ligand concentrations (Fig. 1), but also show classical desensitization patterns upon repeated agonist stimulation (Fig. 5), suggesting that the DMR signal can serve as a novel readout for monitoring receptor activation. Interestingly, the two PAR_2_-specific agonists SLIGRL-amide and SLIGKV-amide-induced maximal responses, measured using both Ca^2+ ^flux and MRCAT assays, were significantly different (Fig. 2b). Such difference may be due to the functional selectivity of G protein signaling by soluble PAR agonists [33].

Three lines of evidences suggest that there are functional interactions between PAR_1 _and PAR_2 _in A431. First, among PAR_1,2 _agonists examined, trypsin resulted in the highest Ca^2+ ^mobilization, while SFLLR-amide led to an intermediate Ca^2+ ^mobilization. Similar trend was also observed in their DMR signals (Fig. 2). Co-stimulation with SFLLR-amide and SLIGKV led to Ca^2+ ^mobilization or a DMR signal that is at the level similar to that induced by trypsin alone. These results suggest that: (i) the soluble PAR_1 _ligand SFLLR-amide may partially activate PAR_2_, and (ii) trypsin may transactivate PAR_1 _through unknown mechanism(s). Secondly, the desensitization patterns, as examined using repeated stimulation with various combinations of PAR agonists, also support the functional interactions between PAR_1 _and PAR_2_. The trypsin-treated cells lost their responsiveness to either PAR agonist examined, but not to bradykinin, while the thrombin-treated cells still respond to trypsin. Thirdly, a PAR_1 _partial agonist YFLLRNP-amide can attenuate the DMR signals induced by thrombin or SFLLR-amide, but not SLIGKV-amide. At 729 μM YFLLRNP-amide totally blocked the DMR signal induced by thrombin, but only partially attenuated those induced by either SFLLR-amide or trypsin. Collectively, these results suggest that both SFLLR-amide and trypsin might activate both receptors.

Although it appears that both receptors elicit G_q _signaling in A431, there is distinct difference in the kinetics of receptor re-sensitization. The prolonged stimulation (~1 hr) with trypsin resulted in complete desensitization of cells to sequential stimulation with thrombin, but partial desensitization to SFLLR-amide, SLIGKV-amide or SLIGRL-amide (Fig. 5). On the other hand, the cells still respond to trypsin, after pre-stimulation with thrombin, SFLLR-amide, SLIGKV-amide, or SLIGRL-amide. These suggest that PAR_2 _resensitizes much faster than PAR_1_. It is known that receptor proteolysis and phosphorylation regulate the activities of PARs through receptor internalization and the inhibition of intracellular signal transduction [9,36,37]. Depending on the cellular context, the recovery of functional receptors at the cell surface could take from tens of minutes to hours [38,39].

The almost identical sensitivity of both trypsin- and thrombin-induced Ca^2+ ^mobilization to cholesterol removal suggests that the cell surface cholesterol level plays an equally important role in regulating the amplitudes of Ca^2+ ^mobilization induced by the activation of both PAR_1 _and PAR_2_. It is known that cholesterol extraction leads to the loss of compartmentalization of PtdIns 4,5-P_2_, and G_q_, two important molecules for PAR signaling [40,41]. The suppression of Ca^2+^mobilization by cholesterol depletion might be a direct result of delocalization of PtdIns and G_q_.

Interestingly, the DMR signals induced by trypsin or thrombin exhibited different dependency on the concentration of mβCD in solution. The pre-treatment of cells with mβCD but not its inactive analog αCD attenuated PAR signaling including Ca^2+ ^mobilization and DMR signals induced by thrombin or trypsin. The partial inhibitory effect of EGF pre-treatment suggests that the transactivation of EGFR by cholesterol depletion may attenuate, directly or indirectly, the N-DMR event mediated by thrombin or trypsin (Fig. 8b and 8c). Since the DMR signal is an integrated cellular response, it is very sensitive to the cellular background [17,18]. The activation of EGFR directly by EGF, or indirectly by cholesterol depletion, could alter the cellular background, thus indirectly impairing the N-DMR event induced by both PAR agonists. Alternatively, the EGFR activation or transactivation could lead to signaling pathway(s) crosstalking with GPCR signaling.

For PAR_1_, the cell surface cholesterol level seems equally regulate both Ca^2+ ^mobilization and DMR signal, because both types of cellular responses induced by thrombin exhibited the same sensitivity to mβCD concentration in solution. Conversely, for PAR_2 _the cell surface cholesterol level appears regulate Ca^2+ ^mobilization and DMR signal differently. Such a difference in sensitivity to mβCD concentration between the trypsin- and thrombin-induced DMR signals suggest that two receptors may involve different cellular mechanism(s) or signaling network interactions.

## Conclusion

The signaling of endogenous PAR_1 _and PAR_2 _in A431 was studied using non-invasive and manipulation-free optical biosensor. The biosensor-manifested DMR signals follow classical receptor biology. Similar to Ca^2+ ^mobilization, the DMR signals are saturable to ligand concentrations; exhibit comparable desensitization patterns in response to repeated stimulation with various combinations of agonists; and are sensitive to cell surface cholesterol level. More significantly, data analysis suggests that the biosensor differentiates the signaling of PAR_1 _and PAR_2 _in A431 under physiologically relevant conditions.

## Methods

### Reagents

Thrombin, trypsin, methyl-β-cyclodextrin (mβCD), A23187, AG1478, α-cyclodextrin (αCD), and epidermal growth factor (EGF) were purchased from Sigma Chemical Co. (St. Louis, MO). Fluo-3 was obtained from Molecular Probes (Eugene, OR). SFLLR-amide, SLIGKV-amide, SLIGRL-amide, bradykinin, TFRGAP, GYPGQV, and YFLLRNP-amide were obtained from Bachem (King of Prussia, PA). All compounds were used as received. Corning^® ^Epic™ 96well biosensor microplates were obtained from Corning Inc (Corning, NY), and cleaned by exposure to high intensity UV light (UVO-cleaner, Jelight Company Inc., Laguna Hills, CA) for 6 minutes before use.

### Cell culture

Human epidermoid carcinoma A431 cells (American Type Cell Culture) were grown in Dulbecco's modified Eagle's medium (DMEM) supplemented with 10% fetal bovine serum (FBS), 4.5 g/liter glucose, 2 mM glutamine, and antibiotics. ~5 × 10^4 ^cells at passage 3 to 8 suspended in 200 μl the DMEM medium containing 10% FBS were placed in each well of a 96well microplate, and were cultured at 37°C under air/5% CO_2 _for ~2 days, followed by ~20 hr starvation through continuously culturing in the serum-free DMEM.

### Fluo-3 Ca^2+ ^mobilization assay

Cells were grown in Costar™ 96well clear cell culture microplates. After starvation, the cells were washed with 1× HBSS (1× regular Hank's balanced salt solution, 20 mM HEPES buffer, pH 7.0) in the presence of 2.5 mM probenicid, and labeled in the same buffer containing 4 μM Fluo-3 for 1 hour at room temperature. The cells were then washed twice with buffer, maintained with 100 μl 1× HBSS containing 2.5 mM probenicid. The assay was initiated by transferring 50 μl PAR agonist solution to the cell plate, and calcium signal was recorded over 6 minutes with a 6 sec interval using HTS7000 BioAssay Reader (PerkinElmer Life Science, Boston, MA). The fluorescent intensity before stimulation was recorded and used as a baseline. The percentage increase in fluorescence intensity after stimulation, relative to the baseline fluorescence, was analyzed and used directly as a measure for the increase of intracellular Ca^2+ ^level induced by PAR agonists.

### Optical biosensor measurements

Corning^® ^Epic™ angular interrogation system with transverse magnetic or *p*-polarized TM_0 _mode was used for all studies. The detailed instrumental setup and assay protocols had been previously described [16-18]. Briefly, all compound solutions were prepared using 1 × HBSS containing minimal amount of dimethyl sulfoxide, while the starved cells were washed and maintained with 100 μl the serum-free DMEM. The cells were then treated with 50 μl 1 × HBSS buffered solution in the absence and presence of a compound, followed by stimulation with ligand solutions. The cellular responses were monitored in real time throughout the assays.

For functional recovery after cholesterol removal with mβCD, the quiescent A431 cells were treated with 5 mM mβCD for 15 minutes to ensure the removal of cell surface cholesterol content, followed by washing the treated cells three times with the medium only. The cells were then maintained with 100 μl the medium, and placed into the optical systems. After incubation for 15 minutes to allow cells reaching reasonably steady state, a 100 μl solution of thrombin at 80 unit/ml was added to each well at specific time. The optical responses were recorded throughout the assays.

### Statistical analysis

Unless specifically mentioned, three replicates were carried out for each measurement or each compound. The standard deviation was derived from these measurements (n = 3). The assay coefficient of variation was found to be typically less than 10%. All dose-dependent responses were analyzed using non-linear regression method with the Prism software (Graph Pad).

## Authors' contributions

YF designed and executed part of experiments, and provided interpretation of results. AMF executed most of the optical biosensor experiments. Both authors read and approved the final manuscript.
